# Adherence, frequency, and long‐term follow‐up of video game‐based treatments in patients with attention‐deficit/hyperactivity disorder: A systematic review

**DOI:** 10.1002/brb3.3265

**Published:** 2023-09-24

**Authors:** Lucía Caselles‐Pina, Aaron Sújar, Alejandro Quesada‐López, David Delgado‐Gómez

**Affiliations:** ^1^ Department of Statistics Universidad Carlos III de Madrid Getafe Spain; ^2^ Department of Psychology Universidad Autónoma de Madrid Madrid Spain; ^3^ School of Computer Engineering Universidad Rey Juan Carlos Móstoles Spain

**Keywords:** attention‐deficit/hyperactivity disorder, cognitive training, review, video games

## Abstract

**Background:**

Attention‐deficit/hyperactivity disorder (ADHD) is a prevalent neurodevelopmental disorder in children and adolescents. Recent studies show that video games have great potential for the treatment and rehabilitation of ADHD patients.

The aim of the present review is to systematically review the scientific literature on the relationship between video games and ADHD, focusing on adherence to treatment, frequency of the intervention, and the long‐term follow‐up of video games in children and adolescents with ADHD.

**Methods:**

The preferred reporting items for systematic reviews and meta‐analyses guidelines were adopted. The review protocol was registered in PROSPERO database. We searched in three databases, PubMed, Medline, and Web of Science to identify studies examining the association between video game interventions in ADHD patients.

**Results:**

A total of 18 empirical studies met the established inclusion criteria. The results showed that video games‐based interventions can be used to improve ADHD symptoms and display high adherence to treatment. In addition, in the studies reviewed, the most common intervention frequency is 30 min three to five times per week. However, there is little evidence from studies with video games showing long‐term effects in patients with ADHD.

**Conclusion:**

Video games are useful and effective interventions that can complement traditional treatments in patients with ADHD.

## Graphical abstract

This article reviews the scientific literature on the relationship between video game‐based treatments and ADHD. The results showed that video games‐based interventions can be used to improve ADHD symptoms and display high adherence to treatment. In addition, in the studies reviewed, the most common intervention frequency is 30 min three to five times per week. However, there is little evidence from studies with video games showing long‐term effects on patients with ADHD.

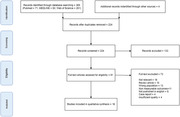

## 1 INTRODUCTION

Attention‐deficit/hyperactivity disorder (ADHD) is the most common psychiatric disorder in children and adolescents, with a worldwide prevalence of 7.2% (Thomas et al., 2015). It is categorized as a neurodevelopmental disorder according to the Diagnostic and Statistical Manual of Mental Disorders, DSM‐5, (American Psychiatric Association, 2013) and presents as persistent inattention, hyperactivity, and impulsivity (Swanson et al., 1998). ADHD can manifest in three different subtypes: inattentive, hyperactive/impulsive, and combined types (Willcutt et al., 2005).

In the long term, people with ADHD have an increased risk of substance abuse, comorbidity with other mental disorders and criminal behavior, as well as a shorter life expectancy (Biederman et al., 2006; Dalsgaard et al., 2014; Young et al., 2016). It can even lead to an eightfold increase in mortality if not adequately treated (Dalsgaard et al., 2015). ADHD also has a profound social, family, and economic impact (Kean, 2005; Pelham et al., 2007).

The treatment of choice for ADHD is multimodal treatment, which consists of a combination of pharmacological treatment and cognitive behavioral therapy (MTA Cooperative Group, 2004). Currently, there seems to be an unresolved problem regarding multimodal treatment. Among the problems that studies have found are low adherence to pharmacological treatment (Gajria et al., 2014; Marcus & Durkin, 2011), low efficacy (Quintero et al., 2018), and even the benefits provided by this treatment disappear with its withdrawal (van de Loo‐Neus et al., 2011). Regarding cognitive behavioral intervention, it is sometimes difficult to access (Castellanos et al., 2002; Culpepper & Mattingly, 2010; Thomas et al., 2015) and has low adherence (Gajria et al., 2014), and some authors question its efficacy (Davis et al., 2018).

To address the difficulties associated with traditional ADHD treatment and improve the quality of life of patients, there are several studies exploring alternative approaches. E‐health could contribute to the management of ADHD in children, as well as help to close the gap in mental health care delivery (O'Dea et al., 2015). A recent area of study is new technologies, such as video games, that can help in multimodal treatment by facilitating the improvement of attention and concentration, favoring collaboration with parents and teachers, and improving the mastery of self‐control, among others (Sánchez et al., 2011).

The importance of video game‐based treatments in the adherence to multimodal treatment of children and adolescents with ADHD should also be considered (Sújar et al., 2022). It seems that the use of video games is more motivating than other types of tools due to the lower number of dropouts (Peñuelas‐Calvo et al., 2022; Rodrigo‐Yanguas et al., 2022). Likewise, the frequency and duration of video game sessions in children and adolescents with ADHD must be considered, as this population is at greater risk of suffering addictions (Menéndez‐García et al., 2022; Sújar et al., 2022). In addition, it is important to monitor the long‐term effect of the use of video game‐based treatments as their short‐term benefit has become clear in several analyses (Cortese et al., 2015; Hodgson et al., 2014; Rapport et al., 2013; Sonuga‐Barke et al., 2013).

Previous reviews have explored the potential of video games for the treatment of children with ADHD (Peñuelas‐Calvo et al., 2022; Rodrigo‐Yanguas et al., 2022; Strahler Rivero et al., 2015). However, there are no systematic reviews that focus on the potential of video games on treatment adherence, the frequency of interventions with video games, and the long‐term follow‐up of their therapeutic benefits.

## 2 AIM

The aim of the present study is to systematically review the scientific literature published to date on the relationship between video games and ADHD considering three elements: (1) adherence to treatment based on dropout rate, (2) frequency of use of video games in the different interventions, and (3) the long‐term treatment follow‐up of the use of video games in patients with ADHD.

## 3 METHODS

This systematic review follows the preferred reporting items for systematic reviews and meta‐analyses guidelines (Moher et al., 2009), and the review protocol was registered as submitted in the PROSPERO database with registration number CRD42023407691.

The PICO research question is as follows: Does video game‐based treatment with a specific frequency increase adherence and long‐term effects in children and adolescents with ADHD?

– Patients: children and adolescents with ADHD,
– Instrument: video game‐based treatment,
– Comparison: control group,
– Outcome: adherence, frequency, and long‐term follow‐up.

### 3.1 Inclusion/exclusion criteria

Inclusion criteria were as follows:

- Studies published in peer‐reviewed journals.
- Studies that include participants under the age of 18 in their sample.
- Studies that tested video game‐based interventions in children with ADHD, providing outcomes about the adherence and/or long‐term follow‐up of such interventions. The diagnosis of ADHD must be confirmed by a clinical specialist or a validated diagnostic tool.
- Studies written in English.
- Studies written in the last 10 years.

Exclusion criteria were as follows:

A. Studies that only reported purely qualitative data.
B. Interventions that target parent/caregivers, teachers, or healthcare providers only.

### 3.2 Search strategy

We proceeded to perform a systematic literature search in three databases: PubMed, Medline, and Web of Science. Last search date was February 2023. The following search terms were used: “(‘video game’ OR ‘video‐game’ OR videogame OR ‘video games’ OR ‘video games’ OR videogames OR ‘video‐games’ OR ‘serious game’ OR ‘computer game’) AND (psychotherapy OR intervention OR rehabilitation OR treatment OR improve* OR enhance OR train*) AND (‘attention‐deficit’ OR ‘attention‐deficit’ OR hyperactivity OR ADHD OR ‘attention‐deficit/hyperactivity disorder’ OR ‘attention deficit/hyperactivity disorder’) NOT (‘internet gaming disorder’).” The references of included studies were also screened.

Articles were selected based on whether they were relevant to the research question, met the inclusion criteria, and were of sufficient methodological quality. An assessment of the quality of the studies was performed using the Newcastle–Ottawa‐scale (NOS; Wells et al., 2000).

Two authors reviewed the studies independently. Inconsistencies in their opinions were resolved with consent. Agreement between reviewers was measured by intraclass correlation coefficient (ICC; Koo & Li, 2016).

## 4 RESULTS

### 4.1 Results of the bibliographical search

In total, 369 articles were selected, of which 145 were eliminated because they were duplicates, and 133 were discarded because they were not related to the purpose of the present review.

Of the remaining 91, 18 were excluded because they were not relevant for this study, 15 were eliminated because they were systematic reviews, 13 were discarded because they used the wrong population for our study, 11 were excluded because they had no measurable outcomes, 8 were eliminated because they were not written in English, 4 were discarded because they were case reports, and 4 were excluded because of insufficient quality.

**FIGURE 1:**
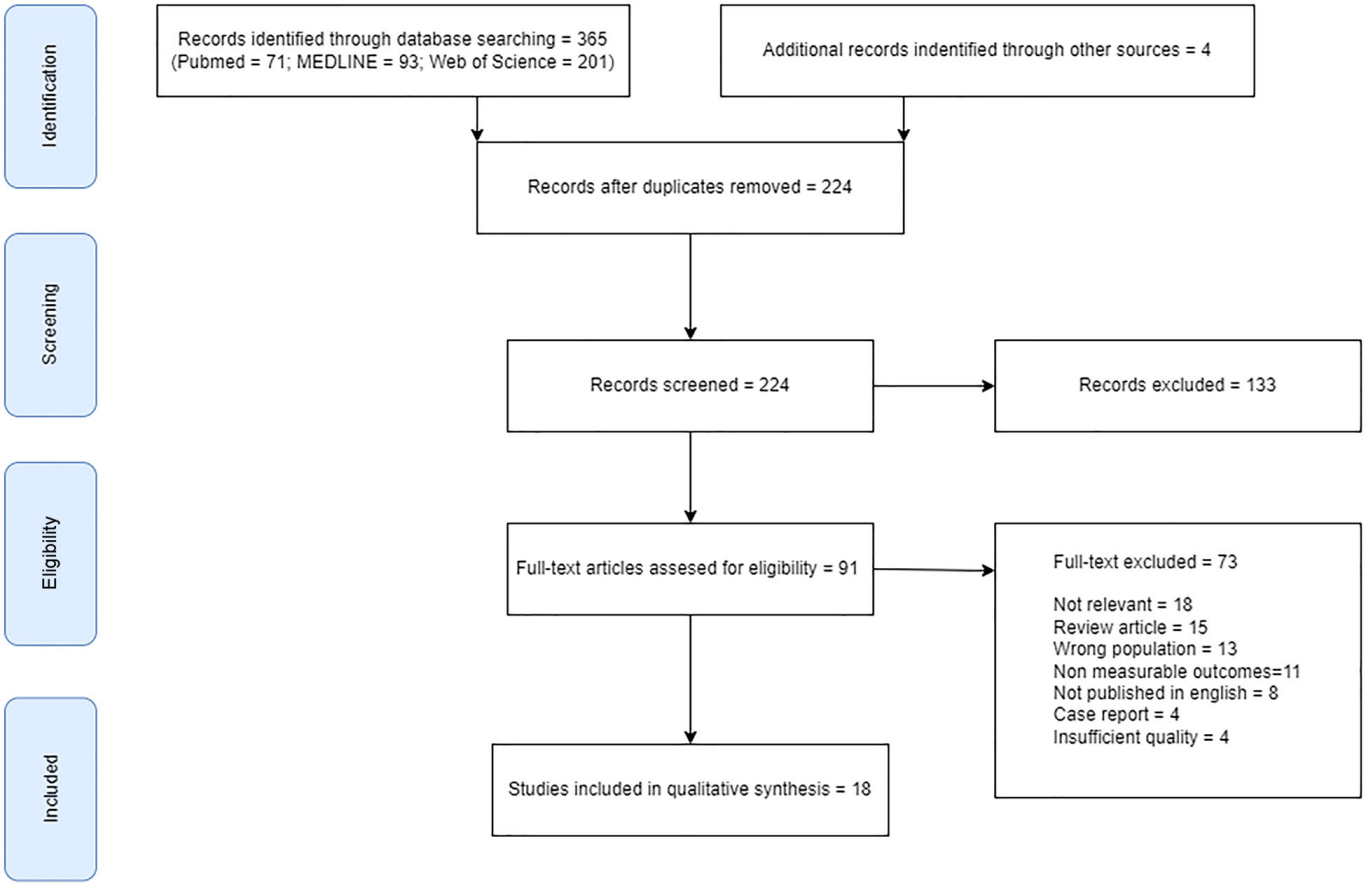
Flowchart of the review following the preferred reporting items for systematic reviews and meta‐analyses (PRISMA) guidelines.

A total of 18 articles were reviewed and included in our systematic review. Figure 1 shows the diagram flow with the decision process.

**FIGURE 2:**
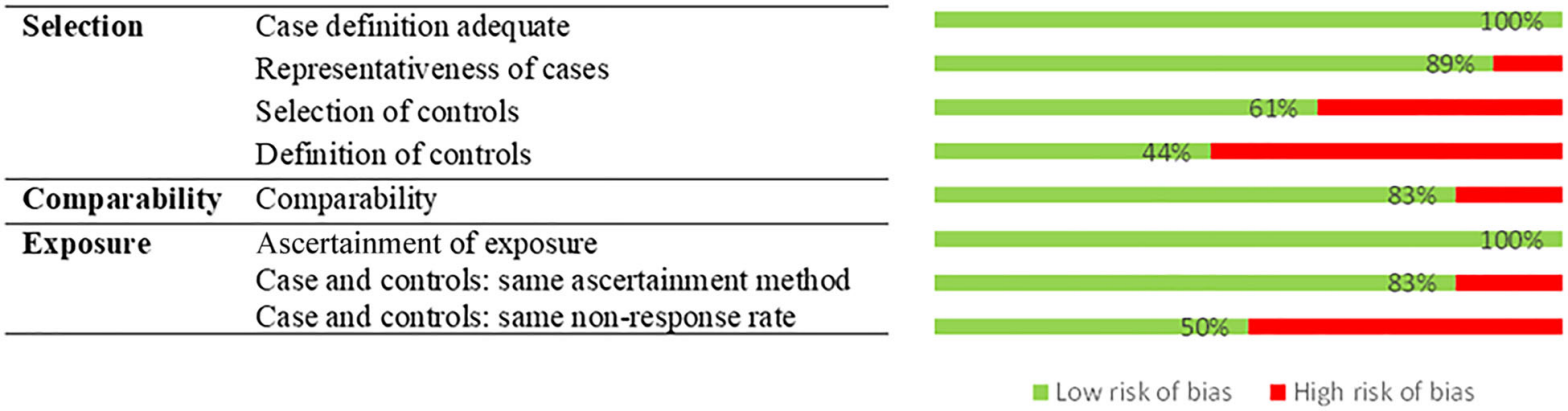
Risk of bias and question in 18 case–control studies using the Newcastle–Ottawa‐scale.

An assessment of the quality of the studies was performed using the NOS. The NOS scores for the studies included in our systematic review ranged from 3 to 8 (range 0–9), with a median and mode of 7. Figure 2 shows the risk of bias assessed by NOS for the selected case–control studies. As can be seen, the selected studies have low risk of bias.

Indicate that all studies obtained one star for adequate case definition and for the ascertainment of exposure. On the other hand, the definition of controls was the item with the lowest number of stars, with 44% of the studies having low bias. This is due to the fact that most of the studies divide the control and intervention groups from a single group of patients diagnosed with ADHD. Finally, the nonresponse rate had a low number of stars. Moreover, we calculated ICC among reviewers, that was 0.923 (95% CI .864–.956) for all articles.

#### 4.1.1 Characteristics of the reviewed studies

Table 1 summarizes the characteristics of the articles that refer to the use of video games as a treatment tool for ADHD. Video game‐based treatments for ADHD can produce significant improvements in attention (Bikic et al., 2017; Davis et al., 2018; Kollins et al., 2021; Lim et al., 2012; Ou et al., 2020; Weerdmeester et al., 2016), hyperactivity and impulsivity (Dovis et al., 2015, 2019; García‐Baos et al., 2019; Kollins et al., 2021; Lim et al., 2012; Ou et al., 2020; Weerdmeester et al., 2016), executive functions (Benzing & Schmidt, 2019; Bikic et al., 2018; Bul et al., 2018, 2016; Dovis et al., 2019; García‐Baos et al., 2019; García‐Redondo et al., 2019; Kollins et al., 2020), working memory (Bul et al., 2016; Chacko et al., 2014; Davis et al., 2018; Dovis et al., 2019, 2015), social skills (Bul et al., 2016), motor skills (Benzing & Schmidt, 2019), and visual skills (Dovis et al., 2015; García‐Baos et al., 2019; Rajabi et al., 2020). On the other hand, some studies (Bikic et al., 2017; Rodrigo‐Yanguas et al., 2021) found no significant differences between the groups tested. For instance, according to Rodrigo‐Yanguas et al. (2021), no differences were found after comparing the combined ADHD subtype with the inattentive subtype. The article by Bikic et al. (2017) also found no significant differences between the intervention group using the video game and the control group using the Tetris game. In this case, the Tetris game improved the patients’ working memory too. In addition, effect sizes are generally medium to large, which supports the idea of the effectiveness of video game‐based treatments. There are a few studies that show effects that are nearly nonsignificant (Bul et al., 2016; Dovis et al., 2019; Rajabi et al., 2020) and others that have effects strongly significant (Bikic et al., 2018; Chacko et al., 2014; Davis et al., 2018; García‐Baos et al., 2019; García‐Redondo et al., 2019; Kollins et al., 2020).

**TABLE 1:** Characteristics of the reviewed studies.

Author	Videogame	Platform	Features	Population (*n*)	% Male	Procedure	Main findings (significance and effect size)
Benzing and Schmidt (2019)	Shape up	Xbox Kinect	Exergame aimed to improve attention, impulsivity, hyperactivity, motor ability, and reaction time	Intervention group (28): mean age = 10.46 (SD = 1.3) Control group (23): mean age = 10.39 (SD = 1.44)	86.4	30 min, three times a week	Intervention group showed faster overall reaction time (*p* = .049, *d* = .58), switching trials (*p* = .029, *d* = .65) and motor ability (*p* = .008, *d* = .80) compared to the control group
Bikic et al. (2017)	Scientific brain training and Tetris	PC	Six different programs aimed to improved different areas of cognition	Intervention group (9) Control group (8) Mean age (all sample) = 15.6 (SD = .99)	76.5	30 min, 5 days a week, for 7 weeks	There are no significant differences between groups on cognition and symptoms of ADHD. Pre–post intragroup measurement showed a significant effect on sustained attention (e.g., *p* = .0026, *d* = 1). The control group had significant effects on working memory (*p* = .0417, *d* = .88)
Bikic et al. (2018)	ACTIVATE	PC	Videogame with different tasks to improve attention, impulsivity, hyperactivity, and reaction time	Intervention group (35): Mean age = 9.77 (SD = 1.97) Control group (35): mean age = 10.14 (SD = 1.52)	84.24	6 days a week for 8 weeks	The intervention had no effect on sustained attention. Intervention group showed greater accuracy in planning (*p* = .006, effect size = 0.30).
Bul et al. (2016)	Plan‐It Commander	PC	Serious game focus on management, planning/organizing and cooperation skills	Intervention group (88): mean age = 9.89 (SD = 1.28) Control group (82): mean age = 9.82 (SD = 1.24)	80.58	A max of 65 min, 3 days a week for 20 weeks	The intervention group compared to control group achieved significantly greater improvements on time management skills (*p* = .004, *d* = .39), social skill of responsibility (*p* = .04, *d* = .04) and working memory (*p* = .02, *d* = .51)
Bul et al. (2018)	Plan‐It Commander	PC	Serious game focus on management, planning/organizing and cooperation skills	Intervention group (88) Control group (82) Mean age (all sample) = 9.90 (SD = 1.26)	82	A max of 65 min, 3 days a week for 20 weeks	Girls showed the greatest improvements in planning/organization skills in comparison to the total group. Boys with lower levels of hyperactivity and higher levels of conduct disorder symptoms showed more improvements in their planning/organization skills in comparison to the total group (*d* = .65)
Chacko et al. (2014)	Cogmed WMT	PC	Training program to improve verbal and no verbal working memory	Intervention group (44): mean age = 8.4 (1.4) Control group (41): mean age = 8.4 (1.3)	77.22	30–45 min, 5 days a week for 25 days	Results showed significantly greater improvements in verbal (*p* = .0050, *d* = .28), and nonverbal working memory storage (*p* = .00009, *d* = 1.17), but no discernible gains in working memory storage plus processing/manipulation
Davis et al. (2018)	Project EVO	Ipad	The game is an attention/memory task and a continuous visuomotor “driving” task	ADHD group (18): mean age = 10.35 (SD = 1.4) ADHD high severity group (22): mean age = 10.2 (SD = 1.26) Non‐ADHD (40): mean age = 10.54 (SD = 1.49)	56.25	30–45 min, 5 days a week for 4 weeks	Results showed significant improvements in attention task (e.g., *p* = .003, *d* = .71), and spatial working memory (e.g., *p* = .014, *d* = .51), for the ADHD group. There was no change for the non‐ADHD group
Dovis et al. (2015)	Braingame Brian	PC	Video game aimed to improve working memory, cognition flexibility, and impulsivity	Intervention group (31): mean age = 10.6 (SD = 1.4) Control group (30): mean age = 10.5 (SD = 1.3) Partially active group (28): mean age = 10.3 (SD = 1.3)	79.77	35–50 min, 25 sessions	The intervention condition showed improvement on visuospatial short‐term‐memory and working memory (*p* < .01). Inhibitory performance and interference control only improved in the intervention group and the partially active condition (*p* < .05). Effect sizes ranged from medium to large
Dovis et al. (2019)	Braingame Brian	PC	Video game aimed to improve working memory, cognition flexibility, and impulsivity	Intervention group (31): mean age = 10.6 (SD = 1.4) Control group (30): mean age = 10.5 (SD = 1.3)	80.32	35–50 min, 25 sessions	Pretraining inhibition (*p* = .042, *R* ^2^ = .049) and cognitive flexibility (*p* = .017, *R* ^2^ = .071) were significant moderators of near transfer, and pretraining working memory (*p* = .046, *R* ^2^ = .040) and cognitive flexibility (*p* = .03, *R* ^2^ = .057) were significant moderators of far transfer
García‐Baos et al. (2019)	RECOGNeyes	PC (eye tracker)	The game was designed as an intervention for training visual attention	Intervention group (14) Control group (14) Mean age (all sample) = 11.05 (SD = 2.54)	64.29	30 min, three times a week for 3 weeks	The intervention group showed an improvement in impulsivity (*p* = .0067), reaction time (*p* < .0001), and fixation gaze control (*p* <.0001). No changes were found in control group
García‐Redondo et al. (2019)	Boogies academy and cuibrain	Mobile and tablets	Games based on Gardner's theory of multiple intelligences	Intervention group (24) Control group (20) Mean age (all sample) = 11.83 (SD = 2.71)	61.36	10 min, 2 days a week, 14 weeks	Results indicated that participants in the intervention group showed significantly higher levels of attention, concentration, and correct responses compared to controls (e.g., *p* = .001, *d* = .21)
Kollins et al. (2020)	STARS‐ADHD	PC, mobile, and tablet	Videogame that improves attention, working memory, and inhibition	Intervention group (180): mean age = 9.7 (SD = 1.3) Control group (168): mean age = 9.6 (SD = 1.3)	71.26	25 min, 5 days a week for 4 weeks	The use of the videogame significantly improved performance attention, mean reaction time during infrequent target stimuli, and response variability in patients with ADHD compared with the control group (e.g., *p* = .0005). The effects from pre‐intervention to postintervention were not different from the control condition
Kollins et al. (2021)	STARS‐ADHD	PC, mobile, and tablet	Videogame that improves attention, working memory, and inhibition in children with ADHD	On stimulants group (130): mean age = 10.6 (SD = 1.75) No stimulants group (76): mean age = 10.5 (SD = 1.82)	74.8	25 min, 5 days a week for 4 weeks	The results showed a change in ADHD related impairment (impairment rating scale [IRS]) after 4 weeks. IRS significantly improved in both groups (on stimulants: *p* < .001; no stimulants: *p* < .001) after 4 weeks. IRS, ADHD rating scale (ADHD‐RS: e.g., *p* < .001, *d* = .74), and clinical global impressions scale (CGI‐I: e.g., *p* < .001, *d* = .81) remained stable during the pause and improved with a second treatment period
Lim et al. (2012)	Cogoland	PC	BCI system where the avatar moves if the participant is focused detected by EEG	Intervention (20): mean age = 7.8 (SD = 1.4)	80	30 min, three sessions a week for 3 months	Significant improvement in ADHD symptoms for inattentive symptoms and hyperactive‐impulsive symptom (both *p* < .01, *d* = .78–.84). Monthly reinforcement did not significantly improve symptoms
Ou et al. (2020)	Fishing master, fruit train, and ocean manager	Virtual reality game console	These videogames train children's attention and cognitive and behavioral performance	Intervention group (3): mean age = 9.67	33.33	40 min (with a 5 min break after each 10 min session), three times a week for 3 months	The results revealed an improvement in their performance in attention, hyperactivity/impulsivity, and oppositional defiance
Rajabi et al. (2020)	SmartMind	PC	These games are based on working memory and inhibitory control training	Intervention group (16): mean age = 10.20 (SD = 1.03) Control group (16): mean age = 10.05 (SD = .83)	100	45 min, three times a week for 3 months	The effect of neurofeedback training on visual performance (visual attention (*p* < .01, Eta = .41) and visual response control (*p* < .05, Eta = .22) was significant. There was no significant effect on auditory attention and auditory response control
Rodrigo‐Yanguas et al. (2021)	The Secret Trail of Moon	PC (VR glasses)	Serious video game designed for cognitive training related to ADHD symptoms and executive dysfunction	Intervention group (37): mean age = 13.78 (SD = 2.28) Combined subtype (21): mean age = 13.38 (SD = 2.16) Inattentive subtype (16): mean age = 14.31 (SD = 2.39)	68	Cognitive training (25 min) and exploring the forest (10 min)	There are no significant differences comparing the ADHD combined subtype to the inattentive subtype
Weerdmeester et al. (2016)	Adventurous Dreaming Highflying Dragon	PC or Xbox 360	Game focusing on inattention, hyperactivity, impulsivity, and motor deficiency	Intervention group (37): mean age = 9.84 (SD = 1.71) Control group (36): mean age = 9.69 (SD = 1.79)	79.45	15 min, six sessions for 3 weeks	The intervention group reported a greater increase in false alarms (impulsivity) than the control group (*p* < .05, Eta = .04–.06)

Abbreviations: ADHD, attention‐deficit/hyperactivity disorder; *d*, Cohen's; Eta, eta squared; *p*, *p*‐value; *R* ^2^, *R*‐square increase due to interaction.

Video games for ADHD treatment may also improve adherence to intervention (Bikic et al., 2018; Dovis et al., 2019). This benefit may be due to the increased motivation provided by video games as they are perceived as enjoyable activities (Rodrigo‐Yanguas et al., 2021) and consequently to the low number of dropouts in the reviewed articles. As can be seen in Table 2, four studies have a dropout rate of less than 10% (Bikic et al., 2017, 2018; Davis et al., 2018; Dovis et al., 2019; Kollins et al., 2020), and six articles have dropout rates between 10% and 20% (Benzing & Schmidt, 2019; Bul et al., 2018, 2016; Dovis et al., 2015; Kollins et al., 2021; Lim et al., 2012). Only one study has dropout rates above 20% (Chacko et al., 2014). The reasons for dropouts are varied in the reviewed studies, including loss of follow‐up, lack of motivation on the part of the children, adverse events like headache (Bul et al., 2016; Kollins et al., 2020; Lim et al., 2012), frustration (Kollins et al., 2020), pain in the fingers or irritability (Bul et al., 2016), or parental withdrawal, among others. Finally, only one study reported a lack of participant dropouts (Weerdmeester et al., 2016).

**TABLE 2:** Dropout, long‐term follow‐up, and adverse effects of the reviewed studies.

Author	Dropout (%)	Long‐term follow‐up	Adverse effects
Benzing and Schmidt (2019)	7 (13.72)	Not reported	Not reported
Bikic et al. (2017)	1 (5.88)	Not reported	No adverse effects
Bikic et al. (2018)	5 (7.14)	The effects of the ability to plan seemed to be sustained over time	No adverse effects
Bul et al. (2016)	31 (18.23)	Time management and working memory skills effects maintained or even further improved at 10‐week follow‐up	Pain in the fingers, irritability, and headache
Bul et al. (2018)	31 (18.23)	Not reported	Not reported
Chacko et al. (2014)	19 (22.35)	Not reported	Not reported
Davis et al. (2018)	4 (5)	Not reported	Not reported
Dovis et al. (2015)	11 (12.36)	Not reported	Not reported
Dovis et al. (2019)	3 (5)	Not reported	Not reported
García‐Baos et al. (2019)	Not reported	Not reported	Not reported
García‐Redondo et al. (2019)	Not reported	Not reported	Not reported
Kollins et al. (2020)	19 (5.45)	Not reported	Frustration and headache
Kollins et al. (2021)	25 (12.13)	Not reported	No adverse effects
Lim et al. (2012)	3 (15)	The behavioral benefits of training at 8 weeks were maintained at 24 weeks (long‐term effect)	Headache
Ou et al. (2020)	Not reported	Not reported	Not reported
Rajabi et al. (2020)	Not reported	Not reported	Not reported
Rodrigo‐Yanguas et al. (2021)	Not reported	Not reported	No adverse effects
(Weerdmeester et al., 2016)	There were no dropouts	Not reported	Not reported

More variability is found in frequency and duration (see Table 1). The duration of video game sessions varies widely across studies and ranges from 10 to 65 min. Specifically, 1 study uses 10 min of gameplay (García‐Redondo et al., 2019), 1 article suggests 15 min (Weerdmeester et al., 2016), 2 studies using the same video game in their analyses suggest 25 min (Kollins et al., 2021, 2020), 11 articles report a range of 30–50 min of gameplay (Benzing & Schmidt, 2019; Bikic et al., 2017; Chacko et al., 2014; Davis et al., 2018; Dovis et al., 2019, 2015; García‐Baos et al., 2019; Lim et al., 2012; Ou et al., 2020; Rajabi et al., 2020; Rodrigo‐Yanguas et al., 2021), and 2 studies use 60–65 min of gameplay (Bul et al., 2018, 2016). Regarding sessions per week, one study employs two sessions per week (García‐Redondo et al., 2019), seven articles use three sessions per week (Benzing & Schmidt, 2019; Bul et al., 2018, 2016; García‐Baos et al., 2019; Lim et al., 2012; Ou et al., 2020; Rajabi et al., 2020), and five studies report five sessions per week (Bikic et al., 2017; Chacko et al., 2014; Davis et al., 2018; Kollins et al., 2021, 2020).

Finally, only three articles have taken long‐term follow‐up into account in their studies (see Table 2). Bikic et al. (2018) exposed the effects of the ability to plan seemed to be sustained over time but do not specify the persistence of these effects. On the other hand, Bul et al. (2016) claimed that time management and working memory skills effects maintained or even further improved at 10 weeks follow‐up. Likewise, Lim et al. (2012) concluded that the behavioral benefits of the 8‐week training were maintained at 24 weeks.

## 5 DISCUSSION

This systematic review suggests that a majority of video game‐based treatments show favorable evidence of being effective in the treatment of ADHD. Previous systematic reviews have reported promising results on video games for ADHD intervention (Peñuelas‐Calvo et al., 2022; Rodrigo‐Yanguas et al., 2022; Strahler Rivero et al., 2015) but to our knowledge, this is the first systematic review that focuses on reviewing adherence, time spent playing sessions, and long‐term follow‐up.

The aim of this systematic review is to provide the research community with knowledge on video games for the treatment of ADHD, such as frequency of the interventions, adherence data, and the long‐term effects of the use of this kind of interventions. The information presented here will be possible to use as a basis for future studies.

Although there is currently a large amount of scientific evidence on treatments with video games in patients with ADHD, several limitations of the articles reviewed in this study have been found, such as the lack of replication of the research or the low sample sizes used.

The information presented in Table 1 shows that video game‐based treatments provide benefits for children and adolescents with ADHD. Access to specialized multimodal treatments for ADHD is limited in some areas (Castellanos et al., 2002; Culpepper & Mattingly, 2010; Thomas et al., 2015). In addition, such treatments are often financially costly for patients and their families and have high discontinuation rates (Jensen et al., 2005; Pottegård et al., 2015). The fact that most of cited video games can be played in any location and at any time may help to reduce the barrier of physical and financial accessibility to treatments and thus facilitate early care and intervention for patients (O'Dea et al., 2015).

Additionally, these tools can help in multimodal treatment by facilitating the improvement of attention and concentration, favoring collaboration with parents and teachers and improving self‐control skills among others (Sánchez et al., 2011). Likewise, the potential of an intervention with exergames (video games with physical activity) is also relevant for counteracting cognitive and motor deficits in children and adolescents with ADHD, as these subjects often dropout of conventional sports programs (Lee et al., 2014). For all these reasons, it would be interesting for video game‐based treatments to serve as a complementary activity to psychoeducational treatment.

Several factors must be considered when creating this type of video game. For starters, the game's level of difficulty must be adjusted to the patient's competence and increased progressively over time, whereas the patient's progression must be made visible to them by means of positive reinforcement at the immediate moment (Shaw & Lewis, 2005). Currently, the tendency is to employ precise gaming, which is a personalized treatment that adapts to the capabilities of the user (Kinross, 2018). Likewise, other secondary aspects such as time management, social interactions, and abilities transferable to daily life situations should be considered (Rodrigo‐Yanguas et al., 2022; Sújar et al., 2022).

On the one hand, the results presented in this review show high adherence to treatment with the use of video games, with most having low dropout rates by participants. Gamification is a technique used in e‐health interventions that promote behavior change and engagement of participants (Hamari et al., 2014). In children, the rewarding effects as well as the use of immediate rewards and the novelty of video game‐based treatments challenges may be of relevance in increasing adherence (Sújar et al., 2022). In addition, young people are usually difficult to engage in interventions, and video games are often not perceived as an imposition by parents or professionals, which may be more interesting and enjoyable for them (Bussing et al., 2012; Tatla et al., 2014). Video games can also increase the participation and motivation (Granic et al., 2014; Prins et al., 2011). This is relevant as research suggests that children with ADHD with motivational problems may have a decrease in the beneficial effects of executive function training (Prins et al., 2011).

The adherence provided by video games as a treatment for ADHD should also be considered to complement other treatments such as pharmacological intervention or cognitive behavioral therapy. Adherence to pharmacological treatment and behavioral therapy is often quite poor (Gajria et al., 2014; Marcus & Durkin, 2011). In a recent review, it was found that in clinical studies, the mean rates of nonadherence to drug treatment ranged from 13.2% to 64% (Adler & Nierenberg, 2010). Antshel and Olszewski (2014) wanted to study the adherence to therapy of a group of adolescents with ADHD and concluded in their study that 45% of the participants stopped attending cognitive behavioral therapy sessions at some point during the intervention. In the present review, these rates are between 5% and 22.35%, therefore confirming that video game‐based treatments seem to have higher levels of adherence. Furthermore, video games can help to increase the level of engagement and adherence to specific treatments such as neurofeedback therapies (Blandón et al., 2016), as these have limitations in terms of usability and adherence due to their long duration of intervention (Chantry & Dunford, 2010).

On the other hand, this study exposes the frequency of playing sessions of video game‐based treatments. This fact is very interesting, because there is not enough information on what the adequate numbers of play sessions and session duration are for the intervention of children and adolescents with ADHD. The frequency of the intervention in most of the reviewed studies in this article is around 30 min three to five times per week. As most of the reviewed studies present video games that provide positive effects on the quality of life of children and adolescents with ADHD, this duration and frequency of sessions seem reasonable and plausible with the fact that these patients often present concentration difficulties and are at risk of addictions (Menéndez‐García et al., 2022; Raniyah & Syamsudin, 2019).

Regarding the frequency and duration of pharmacological and non‐pharmacological treatments, there seems to be no consensus. Pharmacological treatments are medical prescriptions, and as such, there is no clear information about their duration (Zhang et al., 2021). Furthermore, it depends on the follow‐up that the patient is subjected to afterward (van Walraven et al., 2004). Similarly, there also seems to be no consensus on the duration and frequency of psychological interventions such as cognitive behavioral therapy sessions (Cook et al., 2014; Mongia & Hechtman, 2012). However, it should be stressed that just as pharmacological treatment is for continuous use (Wolraich et al., 2007), video games should be a periodic intervention over time and not an isolated training.

Finally, in relation to the long‐term follow‐up of improvements in symptoms with the use of video games in the treatment of ADHD, only three articles have been found that mention this effect. This may be because most of the studies reviewed in this paper have rather short intervention periods. As the use of video games as a therapeutic tool is currently booming, longitudinal studies exploring the long‐term effects of video games will likely take some time to appear.

On the other hand, some meta‐analyses (Cortese et al., 2015; Hodgson et al., 2014; Rapport et al., 2013; Sonuga‐Barke et al., 2013) that executive function training interventions in children with ADHD improve short‐term effects but have very limited long‐term effects. However, studies with longer intervention procedures could evaluate the long‐term effects of video games and thus be able to discern if these tools can benefit people with ADHD to maintain the effects of treatment over a longer period. It is therefore essential to understand which characteristics of games can cause substantial changes in the quality of life of children and adolescents with ADHD (Sújar et al., 2022).

Another reason why it is relevant to know the possible long‐term effects of video games as a treatment for ADHD is to be able to carry over their benefits to other treatments other treatments such as cognitive behavioral therapy or pharmacological intervention. First‐line treatment for ADHD includes pharmacological and non‐pharmacological interventions (cognitive behavioral therapy), which have shown efficacy in the short term (Catalá‐López et al., 2017; Kollins, 2018). Current medications do not cure ADHD, they merely control the symptoms, whereas they are taken and therefore have no long‐term effects. Adding cognitive behavioral therapy better equips children and adolescents with ADHD and their families to cope with problems associated with the disorder (Pelham et al., 2016; Rijo et al., 2015). However, the effects of therapy are often not sustained beyond the treatment period and there may be doubts about its efficacy in improving ADHD symptoms (Coates et al., 2015; Sonuga‐Barke et al., 2013).

As it has been possible to verify, video game‐based treatments are generally effective. Gamification and cognitive training could be the main mechanisms underlying the usefulness and effectiveness of videogame‐based tools (Peñuelas‐Calvo et al., 2022). However, the fact that people with ADHD tend to be more dependent on extrinsic motivation (Mathews et al., 2019) generates some controversy. Children with ADHD tend to have a higher risk of addiction to video games (Menéndez‐García et al., 2022). To avoid this possible addiction to video games, it is important to reach a compromise between strengthening patient care and reducing gaming time.

Men seem to be the most affected by video game addiction (Mathews et al., 2019) and this coincides with the higher prevalence of ADHD in men than in women (Biederman et al., 2002). Research to date suggests that men tend to start playing video games earlier and that women tend to progress faster toward video game addiction (Black et al., 2015). However, this information must be taken with caution; as observed in our review, the proportion of men in the studies is usually higher, and so these possible gender differences will need to be studied further.

In future lines of research, it would be interesting to consider the potential of video games to improve the quality of life of children with ADHD. Healthcare professionals and computer engineers should collaborate with each other to develop video games that achieve the required therapeutic quality and are adapted to healthcare environments. Furthermore, most of the video games mentioned in this study work on a computer, tablet, or game console, and it would therefore be interesting if they could be adapted for use on smartphones, as these devices could be a good opportunity to implement more effective e‐health.

## 6 CONCLUSION

This systematic review has highlighted the importance of video game‐based treatments as an adjunct to traditional treatments. The treatment of ADHD requires specialized clinical, pharmacological, and cognitive behavioral therapy intervention. However, access to those treatments is sometimes limited in some populations, and video games could facilitate this access for patients.

The use of video games for the treatment of ADHD allows for constant training over time and a level of difficulty and motivation adjusted to the patient, which therefore allows for a decrease in ADHD symptoms and an increase in adherence to treatment. Advances in artificial intelligence will help achieve this goal. Additionally, this review offers an estimated frequency of the intervention with video games for children and adolescents with ADHD that can serve as a reference to avoid possible addictions to video games.

Finally, it is considered necessary to continue investigating the possible long‐term beneficial effects of the use of video games as a treatment for ADHD. Although there is very little information to date on this subject, there is some evidence that suggests that improvements in attention, hyperactivity and impulsivity, and executive functions, among others, can be maintained over time with the use of these tools. It will also be necessary to study how to create video games that are adapted to the age of the patient, so that they can effectively support the treatment of ADHD in children and adolescents.

## AUTHOR CONTRIBUTIONS

Conceptualization; methodology: Lucía Caselles‐Pina and Aaron Sújar. Investigation: Lucía Caselles‐Pina and Alejandro Quesada‐López. Writing—original draft preparation: Lucía Caselles‐Pina, Aaron Sújar, and David Delgado‐Gómez. Writing—review and editing: Lucía Caselles‐Pina, Aaron Sújar, Alejandro Quesada‐López, and David Delgado‐Gómez. Project administration: David Delgado‐Gómez. Funding acquisition: David Delgado‐Gómez. All authors have read and agreed to the published version of the manuscript.

## CONFLICT OF INTEREST STATEMENT

The authors declare no conflicts of interest.

### PEER REVIEW

The peer review history for this article is available at https://publons.com/publon/10.1002/brb3.3265.

## Data Availability

No datasets were generated or analyzed during the current study. The review protocol was registered as submitted in the PROSPERO database under registration number CRD42023407691.
